# Supplementary material for: Assessing the Measurement Properties of the Test of Gross Motor Development-3 Using the COSMIN Methodology—A Systematic Review
Source: Behav Sci (Basel). 2025 Jan 13;15(1):62. doi: 10.3390/bs15010062 (PMC11760866; doi:10.3390/bs15010062)
Supplement: Supplementary file 1 [file behavsci-15-00062-s001.zip › behavsci-3367203-supplementary.pdf]

Table S1. COSMIN Definitions of Measurement Properties

Term			Definition
Domain	Measurement property	Measurement property aspect	
Reliability			The degree to which the measurement is free from measurement error
Reliability (extended definition)			The extent to which scores for patients who have not changed are the same for repeated measurement under several conditions: e.g. using different sets of items from the scale (internal consistency); over time (test-retest); by different persons on the same occasion (inter-rater); or by the same persons (i.e. raters or responders) on different occasions (intra-rater)
	Internal Consistency		The degree of the interrelatedness among the items
	Reliability		The proportion of the total variance in the measurements which is due to “true”a differences between patients
	Measurement Error		The systematic and random error of a patient’s score that is not attributed to “true”a changes in the construct to be measured
Validity			The degree to which the scale measures the construct(s) it purports to measure (depressive symptom severity)
	Content Validity		The degree to which the content of the scale is an adequate reflection of the construct to be measured
		Face Validity	The degree to which (the items of) the scale indeed looks as though they are an adequate reflection of the construct to be measured
	Construct Validity		The degree to which the scores of the scale are consistent with hypotheses (for instance with regard to internal relationships, relationships to scores of other instruments, or differences between relevant groups) based on the assumption that the TGMD-3 validly measures the construct to be measured
		Structural Validity	The degree to which the scores of the scale are an adequate reflection of the dimensionality of the construct to be measured
		Hypothesis Testing	Item construct validity
		Cross-cultural validity/measurement invariance	The degree to which the performance of the items on a translated or culturally adapted scale are an adequate reflection of the performance of the items of the original version of the scale.
Responsiveness	Responsiveness		The ability of the scale to detect change over time in the construct to be measured

Table S2. COSMIN Criteria for Assessing Measurement Properties

Measurement property	Rating	Criteria for rating
Structural Validity	+	Classical Test Theory: Confirmatory Factor Analysis: Comparative Fit Index or Tucker-Lewis Index or comparable measure >0.95 OR Root Mean Square Error of Approximation <0.06 or Standardized Root

		Mean Residuals <0.082
	?	Classical Test Theory: Not all information for + reported
	-	Criteria for + not met
Internal consistency	+	At least low evidence for sufficient structural validity AND Cronbach's alpha(s) ⩾ 0.70 for each unidimensional scale or subscale
	?	Criteria for "At least low evidence for sufficient structural validity" not met
	-	At least low evidence for sufficient structural validity AND Cronbach's alpha(s) < 0.70 for each unidimensional scale or subscale
Cross-cultural validity/measurement invariance	+	No important differences found between group factors (such as age, gender, language) in multiple group factor analysis OR no important differential item functioning for group factors (McFadden's R2 < 0.02)
	?	No multiple group factor analysis OR differential item functioning analysis performed
	-	Important differences between group factors OR differential item functioning was found
Reliability	+	Intraclass correlation coefficient or weighted Kappa ⩾ 0.70
	?	Intraclass correlation coefficient or weighted Kappa not reported
	-	Intraclass correlation coefficient or weighted Kappa < 0.70
Measurement error	+	Smallest detectable change or limits of agreement < minimal important change
	?	Minimal important change not defined
	-	Smallest Detectable Change or Limits of Agreement > Minimal Important Change
Criterion validity	+	Correlation with gold standard ⩾ 0.70 OR Area Under the Curve ⩾ 0.70
	?	Not all information for + reported
	-	Correlation with gold standard < 0.70 OR Area Under the Curve < 0.70
Hypothesis testing for construct validity	+	The result is in accordance with the hypothesis
	?	No hypothesis defined (by the review team)
	-	The result is not in accordance with the hypothesis
Responsiveness	+	The result is in accordance with the hypothesis OR area under the curve ⩾ 0.70
	?	No hypothesis defined (by the review team)
	-	The result is not in accordance with the hypothesis OR area under the curve < 0.70

Note:"+" = sufficient; "-" = insufficient; "?" = indeterminate

Table S3. Levels of Evidence for the Measurement Properties of the TGMD-3

Measurement properties	Methodological risk of bias	Inconsistency	Imprecision	Indirectness of evidence	GRADE level (total number of evidence downgrades)
Content validity (relevant and comprehensible)	NO<br>Four studies of adequate methodological quality	NO	NO	NO	High
Structural validity （Bifactor structure）	NO<br>Thirteen studies of adequate methodological quality	Yes (-1)	NO<br>N> 100	NO	Moderate (-1)
Structural validity	NO	Yes (-1)	NO<br>N> 100	NO	Moderate (-1)

（One-factor structure）		Four studies of adequate methodological quality				
Internal consistency		NO<br>Thirteen studies of adequate methodological quality	Yes (-1)	NO<br>N> 100	NO	Moderate (-1)
Cross-cultural validity		N/A	N/A	N/A	N/A	N/A
Reliability	Test-retest	NO<br>Seven studies of adequate methodological quality	NO	NO<br>N> 100	NO	High
	Inter-rater	NO<br>Nine studies of adequate methodological quality	NO	NO<br>N> 100	NO	High
	Intra-rater	NO<br>Nine studies of adequate methodological quality	NO	NO<br>N> 100	NO	High
Measurement invariance		NO<br>Six studies of adequate methodological quality	NO	NO<br>N> 100	NO	High
Hypothesis testing for construct validity	TGMD-2	NO<br>One study of adequate methodological quality	Yes (-1)	NO<br>N> 100	NO	Moderate (-1)
	M-ABC2	NO<br>One study of adequate methodological quality		NO<br>N> 100	NO	
	GYGBT	NO<br>One study of adequate methodological quality		NO<br>N> 100	NO	
Responsiveness		YES (-2)<br>Two studies of inadequate methodological quality	NO	NO<br>N> 100	NO	Low (-2)

Note: N/A = Not applicable, i.e., no included studies assessed that particular measurement property. TGMD-2 = Test of Gross Motor Development-2; M-ABC2 = Movement Assessment Battery for Children-2; GYGBT = German Youth Games ball-throwing distance performance.
